# CETP genetic variant rs1800777 (allele A) is associated with abnormally low HDL-C levels and increased risk of AKI during sepsis

**DOI:** 10.1038/s41598-018-35261-2

**Published:** 2018-11-13

**Authors:** Kelly Roveran Genga, Mark Trinder, HyeJin Julia Kong, Xuan Li, Alex K. K. Leung, Tadanaga Shimada, Keith R. Walley, James A. Russell, Gordon A. Francis, Liam R. Brunham, John H. Boyd

**Affiliations:** 1 Centre for Heart Lung Innovation, University of British Columbia, Vancouver, BC Canada; 2 Experimental Medicine Program, University of British Columbia, Vancouver, British Columbia Canada; 3 Department of Medicine, University of British Columbia, Vancouver, British Columbia Canada

## Abstract

High-density cholesterol (HDL-C) levels are influenced by genetic variation in several genes. Low levels of HDL-C have been associated with increased risk of acute kidney injury (AKI). We investigated whether genetic polymorphisms in ten genes known to regulate HDL-C levels are associated with both HDL-C levels and AKI development during sepsis. Two cohorts were retrospectively analyzed: Derivation Cohort (202 patients with sepsis enrolled at the Emergency Department from 2011 to 2014 in Vancouver, Canada); Validation Cohort (604 septic shock patients enrolled into the Vasopressin in Septic Shock Trial (VASST)). Associations between HDL-related genetic polymorphisms and both HDL-C levels, and risk for clinically significant sepsis-associated AKI (AKI KDIGO stages 2 and 3) were evaluated. In the Derivation Cohort, one genetic variant in the Cholesteryl Ester Transfer Protein (CETP) gene, rs1800777 (allele A), was strongly associated with lower HDL-C levels (17.4 mg/dL vs. 32.9 mg/dL, P = 0.002), greater CETP mass (3.43 µg/mL vs. 1.32 µg/mL, P = 0.034), and increased risk of clinically significant sepsis-associated AKI (OR: 8.28, p = 0.013). Moreover, the same allele was a predictor of sepsis-associated AKI in the Validation Cohort (OR: 2.38, p = 0.020). Our findings suggest that *CETP* modulates HDL-C levels in sepsis. CETP genotype may identify patients at high-risk of sepsis-associated AKI.

## Introduction

During sepsis, lipopolysaccharide (LPS) and other pathogen lipids are not free in plasma, but rather carried in particles found in the lipoproteins high-density, low-density and very low-density cholesterol (HDL-C, LDL-C, and VLDL-C, respectively). Amongst the lipoprotein fractions, HDL-C has the highest affinity for LPS^1^ and other pathogen lipids^2^ and has anti-inflammatory^3^, anti-thrombotic^4^ and endothelial protective properties^5^. HDL-C levels are low in septic shock^6^, and low HDL-C levels are associated with increased hospital mortality^7^. HDL-C can attenuate systemic inflammation^3,8^ and sepsis-induced acute kidney injury (AKI)^9^. Previously, we demonstrated that HDL-C levels drop acutely in sepsis and the magnitude of this drop was a strong predictor of sepsis-associated AKI^10^.

HDL-C levels are strongly influenced by genetics in non-septic patients^11^. Single nucleotide polymorphisms (SNPs) in genes involved in HDL-C metabolism such as *CETP*^12–14^, *ABCA1*^13,14^, *SCARB1*^15^, *APOA1*^16^, *LIPG*^14^, and *LCAT*^14^ among others, have been associated with changes in HDL-C levels in comparison to WT genotype in the healthy population. Moreover, renal function and plasma HDL-C are tightly linked because kidneys clear and control recycling of senescent HDL-C particles while their filtration function appears to be highly associated with the level and content of HDL-C particles^17^.

It is still unclear whether host genotype influences HDL-C levels at the onset of and during sepsis. If the decline in HDL-C causes AKI then variants known to alter HDL-C levels should also result in greater risk of sepsis-associated AKI. Therefore, we hypothesized that genetic variation in genes known to modulate HDL-C metabolism is associated with the development of sepsis-associated AKI. Accordingly, the purpose of this study was to investigate associations between SNP(s) in gene(s) related to HDL-C levels and the risk of sepsis-associated AKI.

## Results

### Derivation Cohort

#### The candidate CETP variant rs1800777 (allele A) was associated with HDL-C levels at sepsis admission

**Figure 1:**
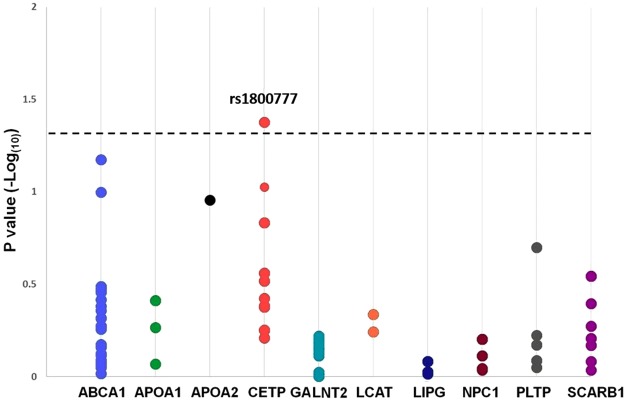
Associations between HDL-C levels at sepsis vs. genetic variations per genes analyzed. Plots represent the corrected P value (Benjamini-Hochberg correction with a false discovery rate cutoff of 0.05) per each variant. X-axis: genetic variations analyzed per gene; Y-axis: P values (−log_(10)_) per each genetic variant; The horizontal dotted line represents the −log_(10)_ for the corrected P value of 0.05. The candidate variant rs1800777 was the only one that showed a statistically significant association with HDL-C levels measured at sepsis admission (P = 0.042). Some plots represent overlapped P values of 2 or more genetic variants.

We tested for association between common (MAF ≥ 1%) variants in HDL-related genes (*ABCA1*, *APOA1*, *APOA2*, *CETP*, *GALNT2*, *LCAT*, *LIPG*, *NPC1*, *PLTP*, and *SCARB1)* and HDL-C levels in patients with sepsis in the Derivation Cohort. Only the minor allele of the CETP variant rs1800777 (allele A) showed a significant association with HDL-C levels at sepsis admission after Benjamini-Hochberg correction (P = 0.042) (Fig. 1). Results of associations between each variant (within each analyzed gene) and HDL-C levels at sepsis admission are shown in Table 1 (Supplementary Material). Accordingly, this CETP variant was selected for subsequent analysis and replication.

CETP variant rs1800777 was in Hardy-Weinberg (HWE) equilibrium (Table 2 in the Supplementary Material). Out of 200 patients, 192 (95.0%) were WT, and 10 patients (5.0%) carried one minor allele of the variant rs1800777. No patient was homozygous for the minor allele of this variant. Both CETP genotype groups - WT and R468Q - had similar baseline demographic, physiological and clinical characteristics (Table 1).

**Table 1:** Patients Baseline Characteristics according to rs1800777 variant (allele A).

Variable	WT (N = 190)	rs1800777 (N = 10)	P value
Age – Median (IQR)	57 (44–68)	63 (52–76)	0.249
Gender (N, % male)	123 (64.7)	7 (70.0)	1.000
Ethnicity – N (% Caucasians)	133 (84.2)	7 (87.5)	0.635
**Comorbidities – N (%)**			
• *COPD*	41 (21.6)	2 (20.0)	1.000
• *CKD*	20 (10.5)	1 (10.0)	1.000
• *Chronic Liver Failure*	37 (20.2)	2 (20.0)	0.412
• *CHF NYHA Class 4*	19 (10.0)	1 (10.0)	1.000
• *Diabetes*	49 (25.8)	4 (40.0)	0.461
• *Hypertension*	69 (36.3)	4 (40.0)	1.000
Statins Use – N (%)	54 (28.4)	4 (40.0)	0.480
APACHE II score – Median (IQR)*	9 (5–14)	15 (8–21)	0.043
**Lab**. Parameters – Median (IQR)*			
• *HGB (g/L)*	116 (96–133)	109 (94–136)	0.802
• *WBC (x103/L)*	9.7 (6.3–14.2)	13.5 (5.3–22.2)	0.380
• *Platelets (x103/L)*	206 (146–289)	152 (108–221)	0.073
• *Lactate (mmol/L)*	1.6 (1.2–2.6)	2.3 (1.0–4.2)	0.695
• *Creatinine (mmol/L)*	85 (65–131)	100 (75–212)	0.342
• *INR*	1.2 (1.0–1.4)	1.2 (1.1–1.7)	0.432

Abbreviations: WT: wildtype; IQR: interquartile range; COPD: chronic obstructive pulmonary disease; CKD: chronic kidney disease; CHF: congestive heart failure; NYHA: New York Heart Association; HGB: hemoglobin; WBC: white blood cells; INR: International Normalized Ratio.

#### CETP variant rs1800777 (allele A) was associated with decreased HDL-C levels and increased CETP mass in sepsis

**Figure 2:**
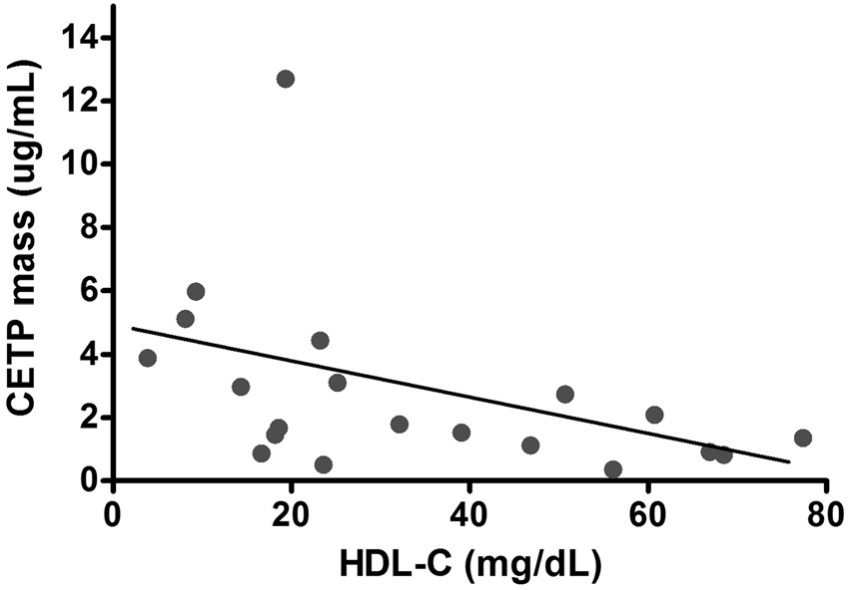
Correlation between HDL-C (mg/dL) and CETP mass (ug/mL). CETP mass and HDL-C measured at sepsis admission showed a statistically significant negative correlation (R^2^ = 0.197, Spearman correlation = −0.555, P = 0.011).

Patients who carried the minor allele of CETP variant rs1800777 had significantly lower median HDL-C levels at sepsis admission in comparison to the WT group (17.40 mg/dL vs. 32.87 mg/dL, P = 0.002) (Table 2). CETP mass was measured in all patients carrying the CETP variant rs1800777 (allele A) (N = 10) and 10 WT patients who were matched for age, sex and ethnicity. The presence of the minor allele A was significantly associated with increased median CETP mass in comparison to WT patients (3.43ug/mL vs. 1.32ug/mL respectively, P = 0.034) (Table 2). Interestingly, CETP mass showed a significantly negative correlation to HDL levels measured at sepsis admission (Spearman’s correlation = −0.555, R^2^ = 0.197, P = 0.011) (Fig. 2).

**Table 2:** Lipids, inflammation and peak of creatinine according to CETP genotype.

Derivation Cohort			
Variable (Median ± IQR)	WT (N = 190)	rs1800777 – allele A (N = 10)	P value
HDL-C (mg/dL)	32.87	17.40	**0.002**
CETP mass (ug/mL)	1.32^*^	3.43	**0.034**
**Validation Cohort (VASST)**			
**Variable (Mean ± SEM)**	**WT (N = 501)**	**rs1800777 – allele A (N = 31)**	**P value**
Peak of sCr (mmol/L)^**^	213 ± 6	296 ± 35	**0.008**
IL-8 (pg/mL)^***^	738 ± 243	1691 ± 1128	**0.049**
IL-6 (pg/mL)^***^	3850 ± 2341	3272 ± 1299	0.133
IL-10 (pg/mL)^***^	373 ± 125	401 ± 175	0.081
TNFα (pg/mL)^***^	23 ± 2	26 ± 7	0.376
MCP-1 (pg/mL)^***^	1576 ± 164	2411 ± 795	0.264

^*^Measurements of 10 patients matched for age, sex and ethnicity; ^**^within 5 days of shock; ^***^available in 284 patients (271 WT patients and 13 patients carrying the CETP rs1800777, allele A).

Abbreviations: WT: wildtype; IQR: interquartile range; HDL-C: high-density lipoprotein-cholesterol; CETP: cholesteryl ester transfer protein; SEM: standard error of the mean; sCR: serum Creatinine; IL: interleukin; TNFα: tumor necrosis factor-alpha; MCP-1: Monocyte Chemoattractant protein-1.

#### CETP variant rs1800777 (allele A) was associated with greater risk of clinically significant sepsis-associated AKI

The frequency of clinically significant AKI was 29.7% (60/202). A higher proportion of patients who carried the minor allele (allele A) of the CETP variant rs1800777 variant developed clinically significant sepsis-associated AKI in comparison to the WT group (70.0% vs. 27.9%, P = 0.009). In our multiple logistic regression model, the presence of the CETP variant rs1800777 (allele A) variant was independently associated with higher risk of development of sepsis-associated AKI stages 2 and 3 (OR = 8.28, P = 0.013). Adjusted and unadjusted logistic regression models are described in (Table 3).

**Table 3:** Adjusted Odds Ratios (aOR)* for development of clinically significant AKI according to CETP genotype (variant rs1800777, allele A) in Derivation and Validation Cohorts.

Cohort	aOR (95% CI)	P value
Derivation	8.28 (1.56–43.78)	0.013
Validation (VASST)	2.38 (1.14–4.95)	0.020

^*^Adjusted for age, sex and ethnicity.

Abbreviations: CETP: cholesteryl ester transfer protein; VASST: vasopressin and septic shock trial.

Interaction analysis among the variants within the CETP gene demonstrated only one strong interaction between rs1800777 and rs5880 (P < 0.001): all patients who carried the variant rs1800777 (N = 10) also carried the rs5880 (N = 18). Patients who carried the latter variant (rs5880) in the absence of the first (rs1800777) showed no statistically significant differences according to frequency or risk of clinically-significant AKI: P = 0.727 (Fisher’s test), and 0.975 (logistic regression), respectively.

#### Causal effect of HDL-C reduction levels on the risk of clinically significant sepsis-associated AKI

**Figure 3:**
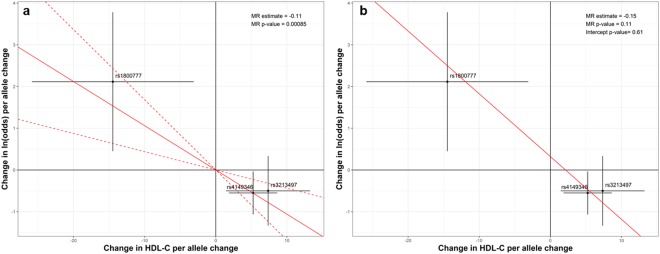
Mendelian Randomization Results. (**a**) Inverse-variance weighting (IVW) analysis including the selected SNPs rs4149346 (*ABCA1)*, rs1800777 (*CETP)*, and rs3213497 (*GALNT2*). X-axis: changes in HDL-C (mg/mL) per allele change; Y-axis: changes in natural logarithm (ln) odds per allele change. Estimated effects on acute kidney injury (AKI) risk are plotted against estimated effects on serum HDL-C for 3 SNPs associated with HDL-C and AKI. IVW estimate: red solid lines; 95% CI: red dashed lines. SNPs associated with increased HDL-C levels decreased the risk of AKI by 11% (ln = −0.11, odds = 0.89) per 1 mg/dL increases in HDL-C, P = 0.00085). (**b**) Pleiotropy Analysis: no unbalanced pleiotropy in the IVW MR analysis was found (P = 0.61) as the intercept was not difference from 0. A negative (but less significant) effect of HDL-C on AKI was demonstrated: the risk of AKI decreased by 14% (ln = −0.15, odds = 0.86) per 1 mg/dL of increases in HDL-C (P = 0.11).

In total, 165 SNPs were tested (out of 216 SNPS, 51 were excluded due to limited frequency of minor alleles in our cohort, defined as MAF ≤ 0.5%) for association with HDL-C levels in the derivation cohort using a linear regression. Ten SNPs in 3 genes (*ABCA1*, *CETP* and *GALNT2*) were found to be significant (P < 0.05). These SNPs were then tested for association with AKI in a logistic regression adjusted for age, sex and ethnicity. To ensure the independence of instrumental variants in the MR analyses, only the SNP that was the most significantly associated with AKI in each of the 3 genes were selected (rs4149346 (*ABCA1)*, rs1800777 (*CETP)*, and rs3213497 (*GALNT2*)). The effect of HDL-C levels on AKI was then evaluated using MR analyses. IVW MR shows that SNPs associated with increased HDL-C levels decreased the risk of AKI (0.89 odds per unit increase in HDL-C, P = 0.00085) (Fig. 3a). The Heterogeneity test shows no significant heterogeneity between the 3 SNPs (P = 0.64). MR-Egger analysis shows the intercept is not significantly different from 0 (P = 0.61), indicating no unbalanced pleiotropy in the IVW MR analysis. MR-Egger shows a negative but less significant effect of HDL-C on AKI (0.86 odds per unit increase in HDL-C, P = 0.11) (Fig. 3b).

### Validation Cohort

#### The association between CETP variant rs1800777 (allele A) and increased risk of clinically significant sepsis-associated AKI was replicated in VASST

Minor allele of the CETP variant was found in HWE (Table 2, Supplementary Material). Baseline characteristics according to CETP rs1800777 genotype (allele A) are described in Table 3 (Supplementary Material). According to CETP genotype, 571 patients (94.7%) were classified as WT while 34 (5.6%) carried the minor allele of the variant rs1800777, including one homozygous patient who was analyzed within the heterozygous group.

Out of 532 patients (after the exclusion of 72 patients with pre-existing CKD), 172 (32.3%) developed clinically significant sepsis-associated AKI. Patients carrying the CETP variant rs1800777 (allele A) had significantly increased frequency of sepsis-associated AKI KDIGO stages 2 and 3 compared to WT patients (51.6% vs. 31.1%, P = 0.030). CETP variant rs1800777 was validated in VASST as the presence of its minor allele A was associated with independently increased risk for developing AKI in our adjusted logistic regression model (OR 2.38, P = 0.020) (Table 3).

#### The CETP variant rs1800777 (allele A) was associated with increased fluid overload and central venous pressure

**Figure 4:**
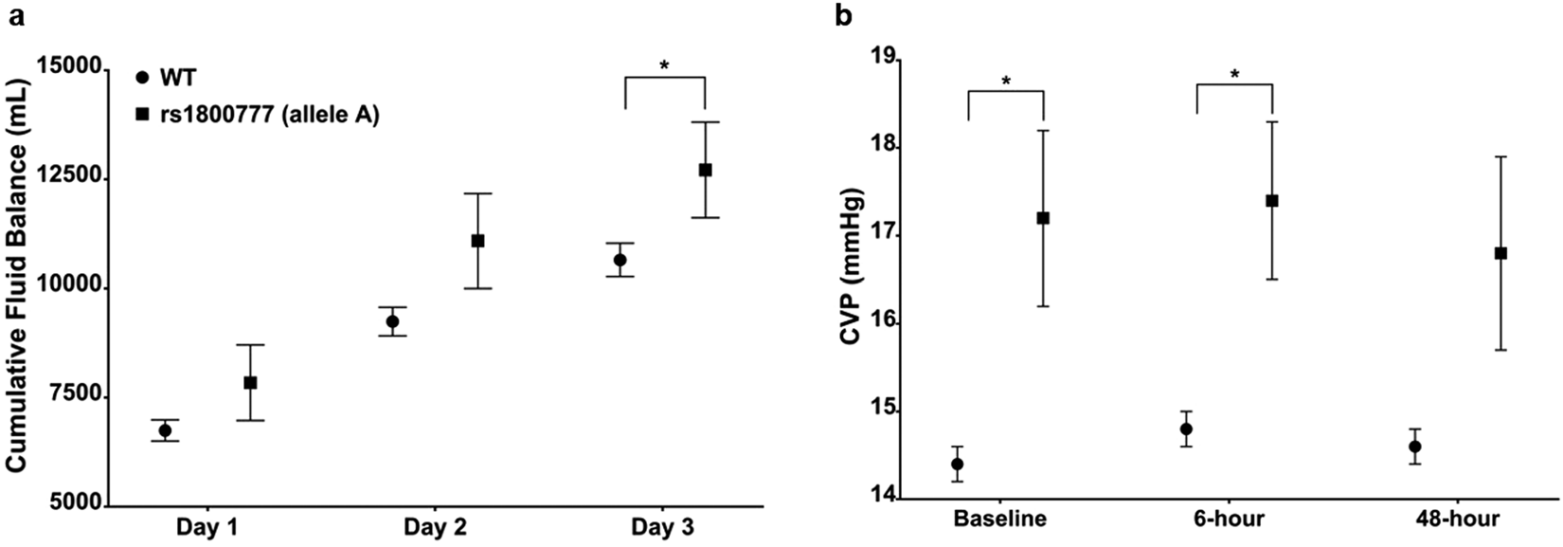
Cumulative Fluid Balance in VASST and Central Venous Pressure (CVP) according to CETP genotype. Patients carrying the CETP variant rs1800777 (allele A) had greater cumulative fluid balance and CVP measurements compared with the WT group. Black circles represent WT patients; Black squares represent patients carrying the CETP variant rs1800777 (allele A). Panel (a) Cumulative Fluid Balance within the first 3 days of shock (D1 to D3); Panel (b) CVP measurements (mmHg) at baseline, 6-hour and 48-hour of shock; *P < 0.05.

Patients carrying the CETP variant rs1800777 (allele A) had significantly greater cumulative fluid balance at D3 (mean cumulative fluid balance at D3 (12,712 mL vs. 10,650 mL, P = 0.049) (Fig. 4a), and higher baseline (17.2 mmHg vs. 14.4 mmHg, P = 0.007) and 6-hour (17.4 mmHg vs. 14.8 mmHg, P = 0.014) CVP compared with the WT group (Fig. 4b).

#### Patients carrying the CETP variant rs1800777 (allele A) presented greater plasma levels of interleukin-8 and peak of creatinine

Increased plasma levels of all 5 cytokines analyzed were found in patients carrying the CETP variant rs1800777 (allele A) in comparison to WT patients. However, only interleukin (IL)-8 resulted in statistically significant differences: 1691 pg/mL vs. 738 pg/mL, respectively, P = 0.049) (Table 2). Similarly, peak of creatinine (mean) during the first 5 days of sepsis was significantly higher in this group of patients when compared to WT (296 mmol/L vs. 213 mmol/L, respectively, P = 0.008) (Table 2).

## Discussion

The major new findings in this study are that the CETP variant rs1800777 (allele A) was associated with a much lower plasma HDL-C levels during sepsis, a great increment in CETP mass, and a doubling of the risk for the development of clinically significant sepsis-associated AKI. By using a meticulous approach for the selection of candidate genetic variant(s), we demonstrated that the CETP variant rs1800777 (allele A) was the only one associated with HDL-C levels in sepsis (out of 10 genes known to affect HDL-C in healthy people)^13^. Most importantly, the association of CETP variant rs1800777 (allele A) and increased risk for sepsis-associated AKI was replicated in the Validation Cohort. We showed a plausible clinical explanation for this finding: CETP variant rs1800777 (allele A) was also associated with increases in cumulative fluid balance, CVP and pro-inflammatory cytokine levels at sepsis, all known risk factors for kidney injury^18^.

CETP mediates the transfer of cholesteryl esters (CE) from HDL-C to Apo-B containing lipoproteins LDL-C and VLDL-C, in exchange for triglycerides (TG)^19^. In normal situations, as this transfer happens, HDL-C becomes richer in TG and more prone to degradation by hepatic and endothelial lipases, which results in a reduction of plasma levels of HDL-C^20^. In accordance with our findings, the CETP variant rs1800777 (allele A) we identify here was previously associated with low HDL-C levels^11^ and high CETP mass^21^. The negative correlation between CETP mass and HDL-C demonstrated by us corroborates the importance of the CETP variant rs1800777 (allele A) for the reduction of HDL levels in patients with sepsis. Therefore, it is biologically plausible that CETP may be a key driver of the acute decline in HDL-C that occurs in sepsis. The first mechanism for the reduction of HDL-C levels in these patients may be mediated by CETP mass elevations that cause greater transfer of CE from HDL to Apo-B lipoproteins, increases in TG-rich HDL amounts, and ultimately increases in HDL-C catabolism by the liver; the second one could be mediated by a prolonged half-life in the mutant form of CETP rs1800777 (allele A)^22^.

Although not proven experimentally in this study, we can speculate how CETP variant rs1800777 (allele A) might cause sepsis-associated AKI. Fluid overload^23^, high CVP measurements^24^ and increased inflammation (IL-8 levels)^25^ are involved in kidney injury and were found in patients carrying the minor allele of the CETP variant rs1800777. This suggests that structural alterations in renal cells caused by fluid overload (e.g., interstitial edema), hemodynamic changes induced by high CVP (increased afterload and reduced renal perfusion pressure), and host immune response (increased levels of pro-inflammatory IL-8) are factors clinically relevant for the association between CETP genotype and risk of sepsis-associated AKI. Also, the exaggerated reduction in HDL-C levels and hence the minimization of its protective properties in sepsis might enhance systemic and renal inflammation and contribute for AKI development.

We believe our findings presented here may be highly translatable to clinical practice. There is an ongoing search for biomarker(s) that can identify patients at risk of AKI, as not all patients with clinical risk factors for AKI in sepsis (e.g., older age, diabetes, hypovolemia) eventually develop the disease. Our results suggest that HDL-C levels, CETP genotype (for rs180077) and CETP mass may all have a role in the identification of those at very high risk for AKI. Also, we can argue that in the group of patients defined at high risk of AKI, CETP can be rapidly inhibited with drugs proven effective in phase 3 clinical trials^26–29^. Although the first CETP inhibitor (Torcetrapib) evaluated in the ILLUMINATE trial was associated with increased mortality rates due to infection and cancer^26^, the subsequent CETP inhibitors (Anacetrapib, evacetrapib, and dalcetrapib) seem to be safe^27–29^. The finding of a novel genetic marker that influences both HDL-C levels and AKI risk in sepsis favors the use of a theranostic approach in critical care as, hypothetically, we could easily monitor HDL-C levels in patients receiving CETP inhibitors, drugs able to raise HDL-C within a relatively short period^26–29^. Preclinical studies are necessary before future clinical trials of CETP inhibition for the treatment/prevention of sepsis-associated AKI.

Our study has several strengths and limitations. The strengths include the candidate variant selection based on genes that influence HDL-C plasma levels^13^, the lipoprotein formerly demonstrated by us as a strong predictor of AKI when present in low concentrations^10^ and regulated by *CETP*^12^. The chances of false-positive results were minimized firstly using a false discovery rate (FDR) cutoff of 0.05 for multiple comparisons and secondly, by the replication in a second cohort. Our findings present a hypothesis for future randomized controlled trials with CETP inhibitors in sepsis-associated AKI.

The retrospective nature of this study is one of its limitations, which although hypothesis-generating, requires further mechanistic studies to determine more clearly association vs. true causality of the CETP variant rs1800777 (allele A) on the development of AKI. We should mention that our Derivation Cohort was relatively small, which when combined with a genetic variant found in low abundance (5%) does present a challenge when interpreting the biological effect. Moreover, we do not know the reasons for pre-sepsis HDL-C measurements nor for prescription of statins. However, use of statins did not alter HDL-C levels during sepsis in this cohort (HDL-C median value 33.83 mg/dL WT vs. 29.19 mg/dL CETP variant, P = 0.078, data not shown). A further limitation is that our validation (VASST) cohort did not have HDL-C levels measured or data about use of statins. We can reasonably assume that the small impact of statins in HDL-C levels in the Derivation Cohort perhaps would be replicated in VASST.

In summary, CETP variant rs1800777 (allele A) is independently associated with increased risk of development of AKI in two cohorts and this effect is possibly mediated by decrements in HDL-C levels. Our findings strongly support the importance of the *CETP* gene as a modulator of HDL-C levels in sepsis. Future studies evaluating the safety and efficacy of CETP inhibitors in sepsis sepsis-associated AKI are warranted.

## Methods

### Study Design

This was a retrospective observational study of two cohorts. Our Derivation cohort consisted of a 202 patient cohort with lipid measures, sequencing of HDL related genes and AKI status. Details of this cohort have been previously published^30^. Our Validation cohort was a larger 604 patient cohort with DNA polymorphisms and AKI status available. Details are as previously published^31^.

### Ethics

The Institutional Review Board at St. Paul’s Hospital (Providence Health Care Research Ethics Board) and the University of British Columbia Clinical Research Ethics Board approved the present study (Research Ethics Board Number: H11-00505). The Vasopressin And Septic Shock Trial (VASST) was approved by the research ethics boards of all participating institutions (27 centers in Canada, Australia, and the United States)^31^ and the University of British Columbia Clinical Research Ethics Board (coordinating center) approved the genetic analysis.

### Patients and Laboratory methods

The Derivation Cohort included 202 adult patients admitted to the Emergency Department (ED) at St. Paul’s Hospital, Vancouver, Canada, from January 2011 to June 2014 who had criteria for the activation of the Institutional Sepsis Protocol by the attending physician. The sepsis protocol activation requires the presence of a clinically defined infection and at least 2 of the following: (i) Temperature >38 °C or <36 °C; (ii) Heart rate >90 beats per minute; (iii) White blood cell count >12,000 per mm^3^ or <4,000 per mm^3^. All patients from the Derivation Cohort gave written informed consent to the use of both their clinical and analytical data. Study identification numbers were assigned to the secured enrolment forms, and clinical data were stored in an ORACLE-based database on a firewalled, RSS-encrypted server at St. Paul’s Hospital. All experimental methods were carried out in accordance with the approved guidelines.

#### Lipid and CETP measurements

A 6-mL EDTA tube of blood was collected at the time of the first clinical blood draw at the ED. Blood was spun at 1800 g for 12 min, and plasma was aliquoted and stored at −80 °C until processing. HDL-C was measured in plasma on the hospital clinical laboratory’s ADVIA 1800 Chemistry System (Siemens, Richmond, Canada). Plasma CETP mass was measured using a CETP enzyme-linked immunosorbent assay (ELISA) kit (Cloud-Clone Corp., China). HDL-C measurements collected previously to the sepsis event were assessed by review of provincial health records and were used for the determination of the difference between pre-sepsis HDL-C and sepsis admission HDL-C.

#### DNA library preparation and sequencing

DNA extraction, DNA concentration determination, and preparation of DNA library were performed as previously described^13^. Sequencing was performed on an Illumina MiSeq instrument in 2 × 151 bp mode using 300 cycle MiSeq Reagent v2 Kits (Illumina) to generate FASTQ files. FASTQ files were analyzed in BaseSpace apps with BWA Aligner v.1.1.4 (BaseSpace Labs) for alignment to the hg19 reference genome, and Enrichment v.3.0.0 (Illumina) for enrichment analyses and variant calls to generate genome.vcf files. We loaded genome.vcf files into VariantStudio 3.0 for variant annotation. Prior to variant annotation variants were filtered for quality scores >30, read depth >15, and minor allele frequency in the literature >1%. Gene variants that were previously associated with HDL-C levels in non-acutely ill patients^13^ (*ABCA1*, *APOA1*, *APOA2*, *CETP*, *GALNT2*, *LCAT*, *LIPG*, *NPC1*, *PLTP*, and *SCARB1)* were analyzed in 200 patients as 2 patients had no DNA available for sequencing and were excluded of this analysis.

The Validation Cohort included 604 septic shock patients enrolled into VASST^31^ who had DNA available. Written informed consent was obtained from all patients, their next of kin, or another surrogate decision maker, as appropriate. Approval, enrollment, and consent in the VASST Cohort have been described previously^31^. Briefly, inclusion criteria were age older than 16 years, presence of septic shock defined by the presence of two or more diagnostic criteria for the systemic inflammatory response syndrome, proven or suspected infection, and hypotension despite adequate fluid resuscitation. Additional measures available in this cohort were peak of serum Creatinine (sCR) within 5 days of shock, cumulative fluid balance (FB) during the first 3 days of care (defined as all oral and intravenous intake recorded on nursing flow sheets minus urine output and/or dialysis net output, available in 522 patients), central venous pressure at baseline, 6 hours and 48 hours (available in 428 patients), and a panel of 5 cytokine levels measured at baseline (available in 284 patients). Methods used for fluid balance calculation and cytokines measurements are described elsewhere^32,33^. Genotyping for the variant(s) identified in Derivation Cohort was performed as previously described^34^ in VASST for replication.

### Defining Sepsis-associated Acute Kidney Injury (AKI)

In both cohorts, AKI was defined and classified according to the Kidney Disease Improving Global Outcomes (KDIGO) guidelines^35^ into stages 1–3 using methods we have previously published^36^. Patients were categorized into two groups: no clinically significant AKI (patients with no sepsis-associated AKI and KDIGO stage 1) and clinically significant AKI (patients with sepsis-associated KDIGO 2 and 3). The primary outcome was the development of clinically significant AKI over the first 5 days.

### Statistical analyses

Results are expressed as median and interquartile range or mean and SEM for continuous variables and absolute number (%) for categorical variables. The Kolmogorov-Smirnov test was used to determine whether continuous variables were normally distributed. Associations of categorical and continuous variables between 2 groups were tested using Chi-square test (or Fisher’s test when appropriate) and unpaired T-test (for variables normally distributed) or Mann-Whitney test (for variables without normal distribution), respectively.

Gene variants of 10 HDL-related genes with minor allele frequency ≥1% were chosen for analysis (N = 216). We tested associations between each gene variant and baseline HDL-C levels using Mann-Whitney test and assuming an additive model of inheritance. To correct for multiple comparisons, we then selected the candidate(s) variant(s) for testing their associations with clinically significant sepsis-associated AKI based on a corrected P value < 0.05 for the association with HDL-C levels, using the Benjamini-Hochberg method with a false discovery rate (FDR) of 0.05, within each gene. VASST patients were genotyped for the variant(s) that were significantly associated with altered HDL-C levels and tested for association of the variant(s) with clinically significant sepsis-associated AKI. Figure 1 (Supplementary Material) depicts the process of gene(s) and variant(s) selection.

To test our hypothesis that variant(s) in the Derivation Cohort were associated with the risk of clinically significant sepsis-associated AKI, and to identify independent predictors for this outcome, univariate and multiple logistic regression models were performed. The multiple model was adjusted for age, sex, Caucasian ethnicity and the candidate variant. Replication analyses were then performed in the Validation (VASST) cohort.

To better define a clinically relevant mechanism of the association of genetic variant(s) with kidney injury, we also evaluated the association of genetic variant(s) with cumulative FB within 3 days of shock, CVP (baseline, 6-hour and 48-hour), and levels of a panel of 5 cytokines measured at baseline in VASST as an exploratory analysis. All analyses were performed using SPSS Statistics version 23.0 for Windows (IBM Corp., Armonk, NY, USA) and statistical significance was set at an α = 0.05 using two-sided P values.

Variants identified to be associated with HDL-C levels in the derivation cohort were employed in Mendelian randomization (MR) analyses to estimate the causal effect of HDL-C levels on the risk of AKI. We used an inverse-variance weighting (IVW) MR approach, which implements an inverse-variance weighted linear regression of the genetic effects with AKI on the genetic effects with HDL-C levels while constraining the intercept to be zero. Heterogeneity of the SNPs was tested. We also used MR-Egger approach to test pleiotropy. MR analyses were performed using the *“MendelianRandomization”* package (v0.2.2) for R (v3.4.2).

## Electronic supplementary material

Supplementary Information

## Data Availability

The data is this study are available from the corresponding author on reasonable request.
