# Supplementary material for: CETP genetic variant rs1800777 (allele A) is associated with abnormally low HDL-C levels and increased risk of AKI during sepsis
Source: Sci Rep. 2018 Nov 13;8:16764. doi: 10.1038/s41598-018-35261-2 (PMC6233165; doi:10.1038/s41598-018-35261-2)
Supplement: Supplementary file 1 — Supplementary Information [file 41598_2018_35261_MOESM1_ESM.doc]

**Electronic Supplementary Material**

**CETP genetic variant rs1800777 (allele A) is associated with abnormally low HDL-C levels and increased risk of AKI during sepsis**

Kelly Roveran Genga, Mark Trinder, HyeJin Julia Kong, Xuan Li, Alex K. K. Leung, Tadanaga Shimada, Keith R. Walley, James A. Russell, Gordon A. Francis, Liam R. Brunham, John H. Boyd

**Table 1.** Associations between each gene variant analyzed and HDL-C levels at sepsis admission.

**Table 2.** Derivation and Validation Cohorts - CETP rs1800777: Minor Allele Frequency (AMF) and Hardy-Weinberg equilibrium (HWE).

**Table 3.** Patients Baseline Characteristics according to CETP variant rs1800777 (allele A) in the Validation Cohort (VASST).

**Figure 1.** Schematic representation of the process of gene(s) and genetic variant(s) selection.

**Table 1. Associations between each gene variant analyzed and HDL-C levels at sepsis admission**

| **Gene** | **Variants** | **P value^*^** | **P value (corrected)^* *^** | **Gene** | **Variants** | **P value^*^** | **P value (corrected)^**^** |
| --- | --- | --- | --- | --- | --- | --- | --- |
| ***ABCA1*** | rs4149346  rs4743763  rs2067484  rs2297404  rs2853579  rs9282540  rs2066717  rs2066714  rs2297399  rs2020927  rs2230806  rs3818689  rs2066715  rs4149336  rs363717  rs75488496  rs2275545  rs2274873  rs2230808  rs2230805  rs41412244  rs41445345  rs73517878  rs10991377  rs116728780  rs41537052  rs7341705  rs34788556  rs112338016  rs2066881  rs12003906  rs41419649  rs557492263  rs4149341  rs1800977  rs9282544  rs9282537  rs33918808  rs34078184  rs41494750  rs2777801  rs4149338  rs1883025  rs3029584  rs111337110  rs2230807  rs41410048  rs75141626  rs60913410  rs73519810  rs1799777  rs1800978  rs2066716  rs2740485  rs41432545  rs73517870  rs2246841  rs60690601  rs2066718  rs1331924  rs4149339  rs41354653  rs35204915  rs35545593  rs78072322  rs4149340  rs2234885 | 0.001  0.003  0.017  0.024  0.035  0.036  0.038  0.039  0.047  0.051  0.055  0.065  0.068  0.080  0.093  0.105  0.122  0.13  0.191  0.193  0.197  0.197  0.203  0.215  0.216  0.216  0.216  0.230  0.231  0.242  0.256  0.319  0.334  0.350  0.362  0.404  0.452  0.454  0.465  0.465  0.476  0.477  0.523  0.554  0.570  0.591  0.591  0.591  0.619  0.623  0.638  0.638  0.669  0.701  0.720  0.720  0.727  0.786  0.787  0.790  0.791  0.841  0.842  0.842  0.842  0.878  0.962 | 0.067  0.100  0.326  0.326  0.326  0.326  0.326  0.326  0.335  0.335  0.335  0.350  0.350  0.382  0.415  0.439  0.480  0.483  0.533  0.533  0.533  0.533  0.533  0.533  0.533  0.533  0.533  0.533  0.533  0.540  0.553  0.667  0.678  0.689  0.692  0.751  0.760  0.760  0.760  0.760  0.760  0.760  0.814  0.822  0.822  0.822  0.822  0.822  0.822  0.822  0.822  0.822  0.845  0.854  0.854  0.854  0.854  0.867  0.867  0.867  0.867  0.867  0.867  0.867  0.867  0.891  0.962 | ***GALNT2*** | rs3213497  rs3748008  rs16851269  rs3213495  rs60775651  rs3811486  rs3811485  rs112499399  rs3811487  rs16851339  rs2273967  rs2273969  rs79130823  rs1043900  rs2273966  rs58148281  rs1043908  rs7022  rs2273968  rs11800118  rs3216809  rs78030300  rs11620  rs17710666  rs16851328  rs72647711  rs2273970  rs72647712  rs1043897  rs3811488  rs1043909  rs3811484  rs6698963  rs12091838  rs1923950  rs3213494  rs3748006  rs10495294  rs678050  rs4846914  rs2273965  rs7544606  rs10005  rs1043944  rs13728  rs3088075  rs3213496  rs1043941  rs13490  rs111823613  rs76813899 | 0.025  0.043  0.050  0.063  0.070  0.071  0.085  0.113  0.120  0.129  0.137  0.174  0.174  0.183  0.187  0.208  0.236  0.264  0.268  0.284  0.289  0.337  0.340  0.350  0.364  0.364  0.367  0.387  0.388  0.395  0.413  0.440  0.451  0.464  0.471  0.507  0.529  0.559  0.568  0.578  0.600  0.605  0.641  0.678  0.678  0.873  0.873  0.931  0.985  0.995  0.995 | 0.603  0.603  0.603  0.603  0.603  0.603  0.619  0.635  0.635  0.635  0.635  0.635  0.635  0.635  0.635  0.663  0.671  0.671  0.671  0.671  0.671  0.671  0.671  0.671  0.671  0.671  0.671  0.671  0.671  0.671  0.679  0.686  0.686  0.686  0.686  0.718  0.729  0.734  0.734  0.734  0.734  0.734  0.760  0.768  0.768  0.947  0.947  0.989  0.995  0.995  0.995 |
| ***APOA1*** | rs5069  rs5076  rs2070665  rs5070 | 0.149  0.194  0.406  0.855 | 0.388  0.388  0.541  0.855 | ***LCAT*** | rs5923  rs13306496 | 0.230  0.572 | 0.460  0.572 |
| ***CETP*** | rs1800777  rs5880  rs11076176  rs5883  rs12720872  rs5886  rs7192120  rs7196174  rs1801706  rs1800774  rs289741  rs9930761  rs5884  rs891144  rs9935228  rs289742  rs891143  rs1532625  rs5882  rs7205804  rs891142 | 0.002  0.009  0.024  0.028  0.105  0.105  0.105  0.105  0.147  0.152  0.159  0.215  0.256  0.294  0.300  0.442  0.498  0.521  0.531  0.534  0.618 | 0.042  0.094  0.147  0.147  0.275  0.275  0.275  0.275  0.303  0.303  0.303  0.376  0.413  0.420  0.420  0.560  0.560  0.560  0.560  0.560  0.618 | ***LIPG*** | rs199879783  rs59866846  rs35816125  rs874565  rs35978968  rs2276269  rs874566  rs2000813  rs34474737  rs3786248  rs3826577  rs3744840  rs3744841  rs35968328  rs9958734  rs3744843  rs2000812  rs58075967  rs3786247 | 0.088  0.105  0.224  0.291  0.302  0.332  0.335  0.341  0.371  0.562  0.601  0.613  0.644  0.658  0.756  0.784  0.856  0.876  0.906 | 0.824  0.824  0.824  0.824  0.824  0.824  0.824  0.824  0.824  0.940  0.940  0.940  0.940  0.940  0.953  0.953  0.953  0.953  0.953 |
| ***NPC1*** | rs6507717  rs2303880  rs2435307  rs145693774  rs1652377  rs116046557  rs7227375  rs8099071  rs6507720  rs1140458  rs12970899  rs1805082  rs3745024  rs7239575  rs58319130  rs61731962  rs73392120  rs9963518  rs74486453  rs1788799  rs1805081  rs117851153  rs55809701 | 0.098  0.117  0.140  0.171  0.176  0.216  0.216  0.285  0.29  0.311  0.362  0.373  0.376  0.381  0.628  0.628  0.628  0.628  0.652  0.671  0.823  0.867  0.922 | 0.625  0.625  0.625  0.625  0.625  0.625  0.625  0.625  0.625  0.625  0.625  0.625  0.625  0.625  0.771  0.771  0.771  0.771  0.771  0.771  0.901  0.906  0.922 | ***SCARB1*** | rs701103  rs5891  rs10396208  rs59809936  rs77740046  rs838898  rs838915  rs4238001  rs58032386  rs10396210  rs5888  rs5892  rs12580323  rs61932577  rs2070242  rs10396211  rs3825140  rs5889  rs838897  rs2293439 | 0.039  0.043  0.100  0.100  0.100  0.100  0.100  0.165  0.181  0.267  0.293  0.384  0.425  0.434  0.556  0.572  0.575  0.763  0.785  0.923 | 0.285  0.285  0.285  0.285  0.285  0.285  0.285  0.402  0.402  0.532  0.532  0.62  0.62  0.62  0.676  0.676  0.676  0.826  0.826  0.923 |
| ***APOA2*** | rs6413453 | 0.111 | N/A | ***PLTP*** | rs3092096  rs441346  rs2294213  rs6017711  rs73306280  rs11086986  rs553359  rs200238866 | 0.025  0.149  0.410  0.421  0.421  0.712  0.717  0.893 | 0.200  0.596  0.673  0.673  0.673  0.819  0.819  0.893 |

**Table 2. Derivation and Validation Cohorts - CETP rs1800777: Minor Allele Frequency (AMF) and Hardy-Weinberg equilibrium (HWE)**

|  | **MAF** | | **HWE** | |
| --- | --- | --- | --- | --- |
| **Cohort** | **Rare Heterozygous (%)** | **Rare Homozygous (%)** | **Chi-square** | **P-value** |
| **Derivation Cohort (N=200)** | 2.50 | 0.00 | 0.13 | 0.72 |
| **Validation Cohort (N=604)** | 2.73 | 0.16 | 0.51 | 0.47 |

**Table 3. Patients Baseline Characteristics according to CETP variant rs1800777 (allele A) in the Validation Cohort (VASST)**

| **Variable** | **WT (N=571)** | **rs1800777 (allele A) (N=34)** | **P value** |
| --- | --- | --- | --- |
| Age – Median (IQR) | 63 (50 – 73) | 61 (48 – 71) | 0.886 |
| Gender (N, % male) | 335 (58.6) | 20 (60.6) | 1.000 |
| Ethnicity – N (% Caucasians) | 481 (84.2) | 25 (75.7) | 0.153 |
| Comorbidities – N (%)   - *COPD* - *CKD* - *Chronic Liver Failure* - *CHF NYHA Class 4* | 101 (17.7)  69 (12.0)  61 (10.7)  45 (7.9) | 4 (11.8)  3 (9.0)  7 (20.6)  2 (5.9) | 0.488  1.000  0.136  1.000 |
| Lab. Parameters – Median (IQR)^*^   - *WBC (x10^3^/L)* - *Platelets (x10^3^/L)* - *Lactate (mmol/L)* - *Creatinine (mmol/L)* | 13.8 (8.1 – 21.0)  157 (86 – 254)  1.8 (0.9 – 3.6)  148 (90- 250) | 10.2 (4.3 – 16.6)  121 (49 – 205)  2.5 (1.5 – 6.4)  223 (140 – 305) | 0.029  0.033  0.012  0.005 |

Abbreviations: WT: wildtype; IQR: interquartile range; COPD: chronic obstructive pulmonary disease; CKD: chronic kidney disease; CHF: congestive heart failure: NYHA: New York Heart Association; WBC: white blood cells.

**
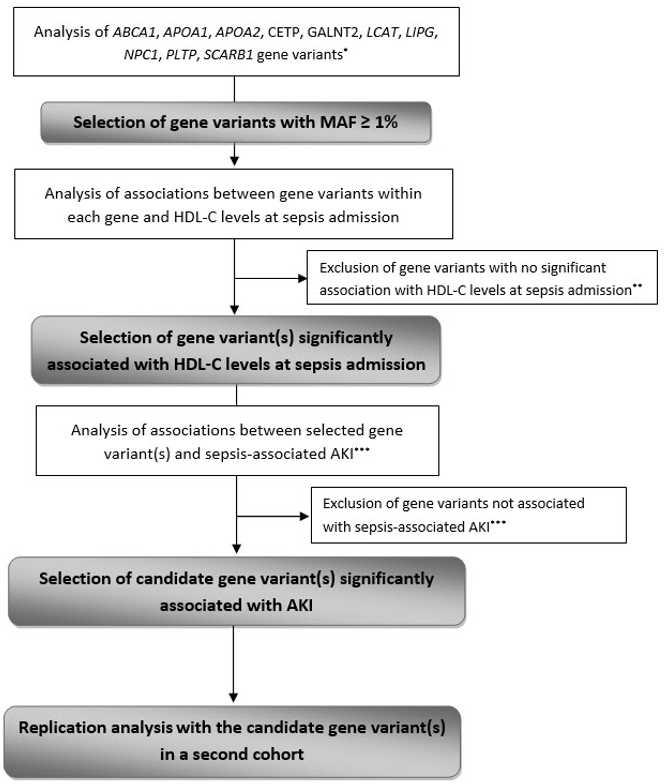
**

**Figure 1. Schematic representation of the process of gene(s) and genetic variant(s) selection.** **^*^**gene variants were chosen based on Sadananda *et al*. J. Lipid Res. 2015. 56: 1993–2001; **^**^**genetic variants were excluded when corrected P value ≥ 0.05 (Benjamini-Hockberg correction within each gene with a false discovery rate cutoff of 0.05); **^***^**clinically significant AKI (AKI Kidney Disease Improving Global Outcomes (KDIGO) = 2 and 3). **Abbreviations:** ABCA1: ATP-binding cassette transporter A1; ApoA1: Apolipoprotein A1; ApoA2: Apolipoprotein A2; CETP: Cholesteryl Ester Transfer Protein; GALNT2: Polypeptide N-Acetylgalactosaminyltransferase 2; LCAT: Lecithin-Cholesterol Acyltransferase; LIPG: Lipase G; NPC1: Niemann-Pick disease type 1; PLTP: Phospholipid Transfer Protein; SCARB1: Scavenger Receptor B1; MAF: Minor Allele Frequency; HDL-C: High-Density Lipoprotein Cholesterol; AKI: Acute Kidney Injury.
